# NT-proBNP and troponin I in acute liver failure: do they predict cardiac dysfunction?

**DOI:** 10.1186/cc9501

**Published:** 2011-03-11

**Authors:** M Mcphail, VK Audimoolam, W Bernal, C Willars, R Sherwood, J Wendon, G Auzinger

**Affiliations:** 1 Imperial College, London, UK; 2 King's College Hospital, London, UK

## Introduction

Distributive shock with high output cardiac failure is frequently seen in acute liver failure (ALF). A previous study suggested a high incidence of myocardial injury coupled with adverse outcome in this population [1]. Correlation of cardiac biomarkers with invasive hemodynamic parameters or results of echocardiographic studies has thus far not been performed.

## Methods

NT-proBNP (NTpBNP) and troponin I (TI) were measured in ALF patients with shock within 48 hours after admission to a tertiary specialist ICU. Transpulmonary thermodilution cardiac output monitoring (PiCCO) was performed in all patients. Values of cardiac index (CI), stroke volume index (SVI), global end diastolic index (GEDI) and markers of contractility - global ejection fraction (GEF) and cardiac function index (CFI) - as well as severity of illness scores were correlated with cardiac biomarker levels. Correlation was assessed using Pearson's coefficient for normally distributed data.

## Results

Twenty-six ALF patients with a mean (SD) APACHE II score of 23 (4) and SOFA 15 (2) were assessed. NTpBNP (median 715 (46 to 10, 484) pg/ml) and TI (median 0.28 (0 to 50) u/l) levels were both significantly elevated without any significant ECHO abnormalities and 24 patients required renal replacement therapy. Serum NTpBNP correlated with serum lactate (correlation coefficient 0.61, *P *= 0.001) and TI (0.63, *P *= 0.001) but not with PiCCO parameters related to flow, contractility or preload (SVI -0.28, *P *= 0.161, CI -0.08, 0.695, GEF -0.26, 0.200, GEDI -0.32, 0.12) and neither cardiac marker correlated with APACHE II score. There was a trend toward correlation of TI with CFI (0.367, *P *= 0.084) but not with CI (0.021, 0.92). CFI was correlated with GEF (0.55, *P *= 0.001) and lactate (0.53, *P *= 0.003). APACHE and SOFA did not correlate significantly with PiCCO indices.

## Conclusions

Levels of cardiac biomarkers are frequently elevated in ALF. We could not find any correlation of TI and NTpBNP with surrogate markers of cardiac function on invasive hemodynamic monitoring, or indeed significant abnormalities on ECHO.
